# Identification and Validation of a Lipid Metabolism-Related Gene Signature for Predicting Prognosis and Immunotherapy Response in Oral Squamous Cell Carcinoma

**DOI:** 10.3390/metabo16070455

**Published:** 2026-06-28

**Authors:** Yu Xie, Ziying Chen, Zhen Chen, Yiming Yang

**Affiliations:** 1 Department of Basic Oral Medicine, School and Hospital of Stomatology, Guangdong Engineering Research Center of Oral Restoration and Reconstruction, Guangzhou Key Laboratory of Basic and Applied Research of Oral Regenerative Medicine, Guangzhou Medical University, Guangzhou 510182, China; 2 Department of Child Healthcare, Shenzhen Bao’an Women’s and Children’s Hospital, Shenzhen 518101, China

**Keywords:** oral squamous cell carcinoma, lipid metabolism, prognostic model, risk score, immunotherapy response, drug sensitivity

## Abstract

**Background/Objectives:** Lipid metabolism plays a critical role in tumor progression and immunotherapy efficacy in oral squamous cell carcinoma (OSCC). However, clinically applicable lipid metabolism-based models for predicting prognosis and immunotherapy response remain limited. This study aimed to develop and validate such a model in OSCC. **Methods:** Using transcriptomic data of OSCC from the TCGA database and a set of lipid metabolism-related genes (LMRGs), we constructed an LMRG-based risk score model via LASSO regression to predict patient survival. This model was subsequently validated using the independent GEO dataset GSE41613. **Results:** Patients in the high-risk group exhibited significantly poorer overall survival than those in the low-risk group (training cohort: *p* < 0.0001; validation cohort: *p* = 0.0086). We also developed a nomogram incorporating the risk score and clinical characteristics, and the risk score was identified as an independent prognostic factor for OSCC patients. Furthermore, the risk score was significantly associated with the tumor immune microenvironment; samples with a lower risk score showed elevated CD8^+^ T cell infiltration and a better response to immunotherapy. Additionally, the high-risk group exhibited an increased tumor mutation burden and resistance to most chemotherapeutic agents. Notably, several drugs (e.g., obatoclax mesylate) showed significant efficacy in the high-risk group, representing promising therapeutic candidates. **Conclusions:** This study reveals that the LMRG signature could serve as a valuable tool for prognosis assessment, risk stratification, and therapy guidance in OSCC.

## 1. Introduction

Oral squamous cell carcinoma (OSCC) constitutes the vast majority of oral cancers, representing over 90% of diagnosed cases [1,2]. Although surgical techniques, radiotherapy, and chemotherapy have advanced over the past decades, the prognosis of OSCC patients has not substantially improved [3,4,5,6]. Local recurrence, lymph node metastasis, and resistance to treatment remain common, all of which contribute to unfavorable survival outcomes [6]. In this context, reliable molecular markers that enable accurate risk stratification are urgently needed to predict prognosis and guide treatment selection in OSCC [7,8].

Metabolic alterations serve as a cancer hallmark, enabling tumor cells to adapt to unfavorable microenvironments and maintain accelerated proliferation [9,10,11]. Among the metabolic alterations reported in tumors, dysregulated lipid metabolism has received increasing attention [12,13]. Tumor cells actively enhance lipid generation, influx, and remodeling to support membrane biogenesis, energy production, and signal transduction [14,15]. For example, activation of the PI3K/Akt/mTOR pathway promotes fatty acid and cholesterol biosynthesis and thereby fuels tumor growth [15]. Dysregulation of lipid metabolism is also linked to signaling pathways governing proliferation, metastasis, autophagy, and ferroptosis [14,16]. These findings indicate that altered lipid metabolism serves functions beyond canonical metabolic roles and can actively contribute to tumor progression.

Lipid metabolism is also implicated in the tumor immune microenvironment (TIME). Lipid metabolites can modulate the differentiation, activation, and function of tumor-infiltrating immune cells [17]. Accumulating evidence indicates that dysregulated lipid metabolic pathways promote the infiltration of immunosuppressive cells, including myeloid-derived suppressor cells (MDSCs), thereby facilitating immune evasion [18,19]. For instance, aberrant fatty acid uptake, mediated by the translocase CD36, can perturb immune signaling and attenuate anti-tumor immune responses [18,20,21]. Collectively, these studies suggest that lipid metabolism can influence both intrinsic tumor cell growth and TIME remodeling, with implications for immunotherapy response.

Although the link between lipid metabolism and cancer has been increasingly recognized, its prognostic value and immune relevance in OSCC remain poorly understood [20]. Existing studies have largely focused on individual genes or specific pathways, whereas systematic analyses of lipid metabolism-related gene signatures in OSCC are still lacking [20,22]. Therefore, constructing a prognostic model based on lipid metabolism-related genes (LMRGs) may provide a more comprehensive understanding of OSCC progression as well as improve risk stratification.

**Figure 1:**
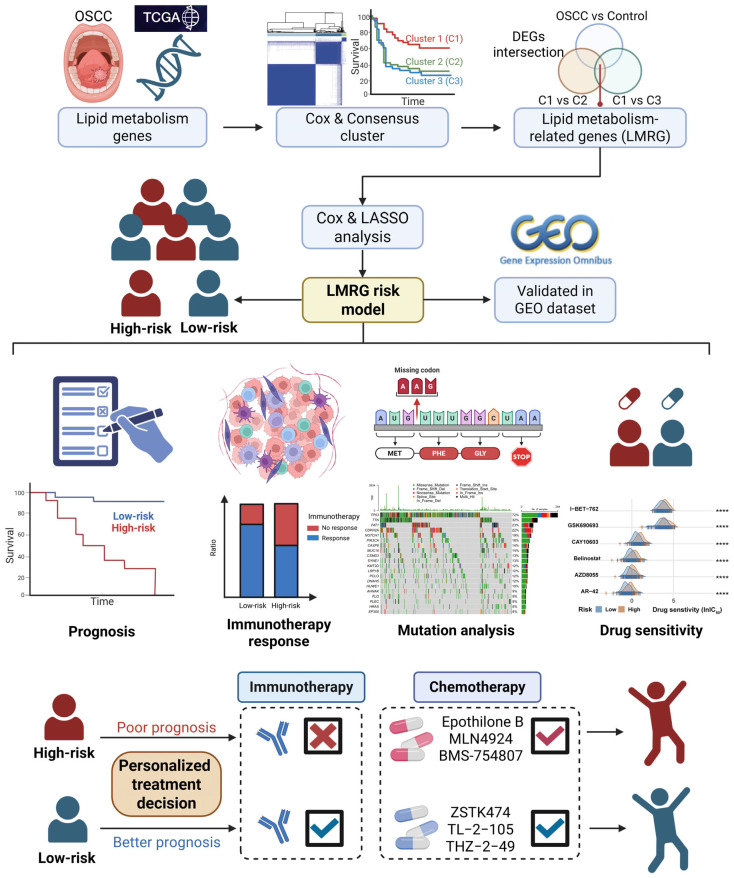
Study workflow for developing an LMRG signature in OSCC. TCGA–OSCC samples were clustered by lipid metabolism gene expression and survival. Intersection of differentially expressed genes across subtypes yielded LMRG, which were used to build a Cox–LASSO prognostic model. High- and low-risk subgroups were compared for prognosis, immunotherapy response, mutation profiles, and drug sensitivity. These results are integrated to guide personalized treatment decisions: high-risk patients may benefit from specific chemotherapeutic agents (e.g., Epothilone B and MLN4924), while low-risk patients may respond to immunotherapy and alternative chemotherapeutics (e.g., ZSTK474 and TL-2-105). The illustration was created with BioRender (https://www.biorender.com/, accessed on 23 April 2026). **** *p* < 0.0001.

In this study, we identified OSCC-associated LMRG and constructed an LMRG-based risk score model using publicly available transcriptomic data. We then assessed and validated the model’s prognostic performance, and analyzed its associations with TIME and therapeutic response. Our findings could help clarify the role of lipid metabolism in OSCC and provide a foundation for prognostic evaluation and individualized treatment (Figure 1).

## 2. Materials and Methods

### 2.1. Data Collection and Preprocessing

RNA-sequencing data and corresponding clinicopathological information for 290 patients with OSCC were retrieved from The Cancer Genome Atlas (TCGA) database (https://portal.gdc.cancer.gov/, accessed on 20 February 2026) as the discovery cohort. Patient selection was restricted to those with primary tumors located at oral-related sites, including base of tongue, floor of mouth, hard palate, lip, oral cavity, and oral tongue (Table S1). Of these 290 patients, two lacked survival data and were excluded from subsequent survival analyses, resulting in a final cohort of 288 patients. For external validation, the GSE41613 dataset, comprising 92 OSCC samples, was obtained from the Gene Expression Omnibus (GEO) database. A comprehensive list of 776 lipid metabolism genes was curated from the Molecular Signatures Database (MSigDB) v7.0 (Table S2).

### 2.2. Subtyping of OSCC Based on Lipid Metabolism Gene Expression

To identify distinct lipid metabolism patterns in OSCC, univariate Cox regression analysis was first applied to screen for lipid metabolism genes significantly associated with overall survival (OS). Subsequently, unsupervised consensus clustering was conducted using the “ConsensusClusterPlus” R package based on these OS-associated lipid metabolism genes. The optimal cluster number was determined according to several established criteria where the cumulative distribution function (CDF) curve ascended gradually and smoothly until reaching a plateau.

### 2.3. Construction and Validation of an LMRG-Based Risk Model

Differentially expressed genes (DEGs) between the identified clusters were extracted via the “edgeR” R package, with filtering criteria of |log_2_FC| > 1 and adjusted *p* < 0.05. Concurrently, DEGs between OSCC and normal tissues were identified following the same criteria. The intersection of cluster-specific DEGs and OSCC-associated DEGs was defined as the final LMRG set. Least absolute shrinkage and selection operator (LASSO) regression analysis was then applied to minimize overfitting and select candidate genes from the LMRG set for the risk model. Patient-specific risk scores were computed according to the following equation:

$$Risk\;\mathrm{score}=\sum \left(Expression_{i}\times Coefficient_{i}\right)$$

Patients were stratified into high-risk and low-risk groups according to the median risk score. Prognostic differences between the high-risk and low-risk groups were assessed by Kaplan–Meier survival analysis and ROC curve analysis. The model robustness was further validated in the independent GEO cohort GSE41613 by evaluating survival outcomes and ROC performance.

### 2.4. Assessment of Clinicopathological Association and Prognostic Value of the Risk Score

Correlation of the risk score with clinicopathological features (e.g., survival status, T stage, grade) was analyzed using the Wilcoxon rank-sum test. The prognostic value of the risk score was further assessed using univariate and multivariate Cox regression analyses. Furthermore, a prognostic nomogram integrating the risk score and clinical parameters was constructed using the “rms” R package to predict 1-, 3-, and 5-year survival probabilities.

### 2.5. Characterization of the Tumor Immune Microenvironment Across Risk Groups

To characterize the immune landscape of OSCC, estimate scores (encompassing immune scores and stromal scores) and tumor purity were calculated for each sample using the ESTIMATE algorithm. The relative abundances of 22 tumor-infiltrating immune cell types were estimated via the CIBERSORT algorithm, and their associations with the LMRG risk score were subsequently examined. In addition, tracking tumor immunophenotype (TIP), immunophenoscore (IPS) and tumor immune dysfunction and exclusion (TIDE) scores were calculated to predict the clinical efficacy of immunotherapy and to assess the sensitivity of patients across risk groups to immune checkpoint inhibitors. The association between the risk score and expression of immune-related molecules was evaluated.

### 2.6. Genomic Mutation Analysis

Somatic mutation data of OSCC patients were retrieved from the TCGA database. The “maftools” R package was employed to visualize the tumor mutation burden (TMB) and mutation landscape of OSCC. The correlation between TMB and the LMRG risk score was analyzed. Waterfall plots were constructed to illustrate the mutation profiles in different risk subgroups.

### 2.7. Drug Sensitivity Analysis

Commonly used chemotherapy drugs for OSCC were retrieved from the Genomics of Drug Sensitivity in Cancer database (https://www.cancerrxgene.org, accessed on 20 February 2026). The log-transformed half-maximal inhibitory concentration (lnIC_50_) values for each drug were estimated using the “pRRophetic” R package. Differences in predicted drug sensitivity between different risk subgroups were evaluated, and the correlation between lnIC_50_ values and the LMRG risk score was further analyzed.

### 2.8. Statistical Analyses

Statistical analyses were performed using R software (v4.2.2). Group differences were assessed using Wilcoxon or *t*-tests. Correlations were analyzed via Spearman or Pearson methods. Survival outcomes were compared using Kaplan–Meier analysis with log-rank testing. Statistical significance was set at *p* < 0.05.

## 3. Results

### 3.1. Identification of Lipid Metabolism Subtypes in OSCC

**Figure 2:**
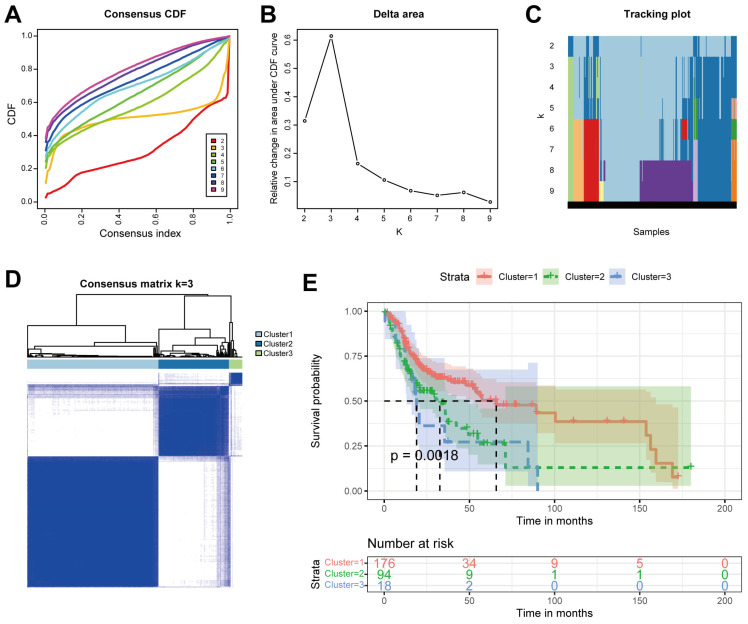
Identification of lipid metabolism subtypes in OSCC. (**A**) CDF curve, (**B**) CDF delta area curve, and (**C**) tracking plot in TCGA–OSCC cohort. (**D**) Consensus matrix heatmap for k = 3. (**E**) Kaplan–Meier curves for overall survival.

Of the 776 lipid metabolism genes retrieved from MSigDB, 71 genes were significantly associated with OSCC survival (*p* < 0.05, Table S2). Based on the expression patterns of these 71 genes, OSCC samples were clustered using the ConsensusClusterPlus R package. At k = 3, the CDF curve exhibited the smoothest profile, with the relative change in area under the CDF curve reaching an inflection point (Figure 2A,B). The tracking plot in the TCGA–OSCC cohort confirmed stable sample clustering at k = 3, and the consensus matrix heatmap further verified the clear separation of three clusters (Figure 2C,D). Thus, k = 3 was selected as the optimal clustering number. Kaplan–Meier survival analysis revealed distinct prognostic outcomes among the three clusters, with Cluster 1 (C1) exhibiting significantly better survival than Cluster 2 (C2) and Cluster 3 (C3) (Figure 2E).

### 3.2. Construction and Validation of the LMRG Risk Model

**Figure 3:**
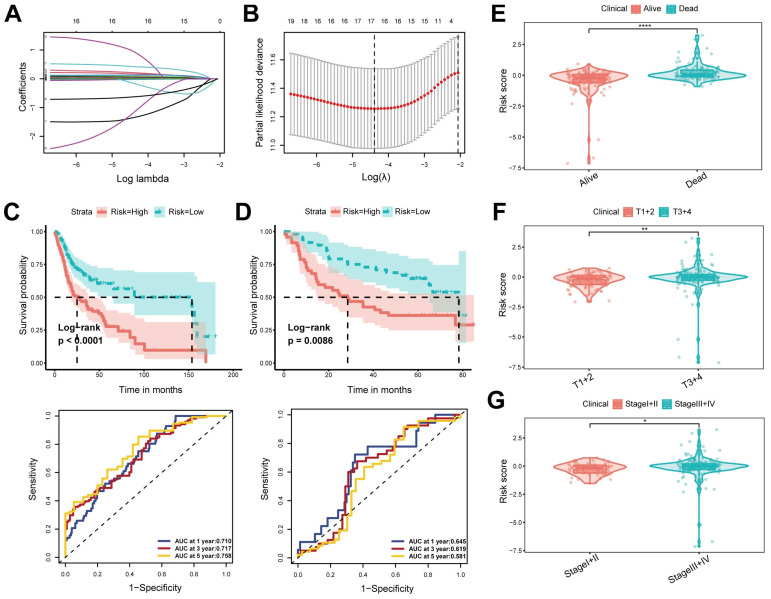
Construction and validation of the LMRG risk model. (**A**,**B**) LASSO regression analysis with optimal lambda. (**C**) Kaplan–Meier curves for overall survival and time-dependent ROC curves evaluating the prognostic value of the LMRG model in the TCGA training cohort. (**D**) Kaplan–Meier curves for overall survival and time-dependent ROC curves evaluating the prognostic value of the LMRG model in the GEO validation cohort. (**E**–**G**) Risk score distribution across survival status, T classification, and clinical stage. * *p* < 0.05, ** *p* < 0.01, and **** *p* < 0.0001.

Given the pronounced differences between C1 and C2/C3 but relatively minor distinction between C2 and C3, we identified DEGs between C1 and C2 as well as DEGs between C1 and C3 using the edgeR package. Intersection of these DEGs with those between OSCC and normal tissues yielded 1248 genes (referred to as LMRG). Subsequently, univariate Cox analysis of LMRG was performed in the TCGA–OSCC training set. A total of 19 genes with *p* < 0.01 were retained and subjected to LASSO regression analysis (Figure 3A,B). Finally, 16 core genes with corresponding coefficients were identified, namely *ADPRHL1*, *ALX1*, *CHRNB4*, *CTLA4*, *GAST*, *GTSF1L*, *HOGA1*, *IL1A*, *LINC01281*, *PCDHA10*, *PCOLCE2*, *POU6F2*, *SLC18A3*, *SLC5A12*, *VWCE*, and *ZNF662.*

Based on the expression levels and coefficients of these 16 genes, a risk score formula was constructed as follows: Risk score = exp(*ADPRHL1*)∗0.066141501 + exp(*ALX1*) ∗0.032014229 + exp(*CHRNB4*)∗0.082662123 + exp(*CTLA4*)∗(−0.025809079) + exp(*GAST*)∗0.013243796 + exp(*GTSF1L*)∗(−1.345376114) + exp(*HOGA1*)∗0.163848745 + exp(*IL1A*)∗0.002858387 + exp(*LINC01281*)∗(−0.164346458) + exp(*PCDHA10*)∗0.937537634 + exp(*PCOLCE2*)∗0.009796966 + exp(*POU6F2*)∗0.209671909 + exp(*SLC18A3*)∗0.12457801 + exp(*SLC5A12*)∗0.41987807 + exp(*VWCE*)∗(−1.16360206) + exp(*ZNF662*)∗(−0.627025493)

High- and low-risk groups were defined based on the median risk score. In the TCGA–OSCC training cohort, Kaplan–Meier curves revealed that the low-risk group exhibited significantly prolonged survival (Figure 3C). Consistent results were observed in the GEO validation cohort (Figure 3D), indicating robust performance of this risk score model in assessing OSCC prognosis. Time-dependent ROC curves at 1, 3, and 5 years yielded area under the curve (AUC) values of 0.710, 0.717, and 0.758, respectively, in the training cohort, indicating that the model has satisfactory prognostic accuracy for risk stratification in OSCC patients (Figure 3C). In the validation cohort, AUCs were 0.645, 0.619, and 0.581, respectively (Figure 3D).

### 3.3. Characterizing the Clinical Relevance of the Prognostic Model

**Figure 4:**
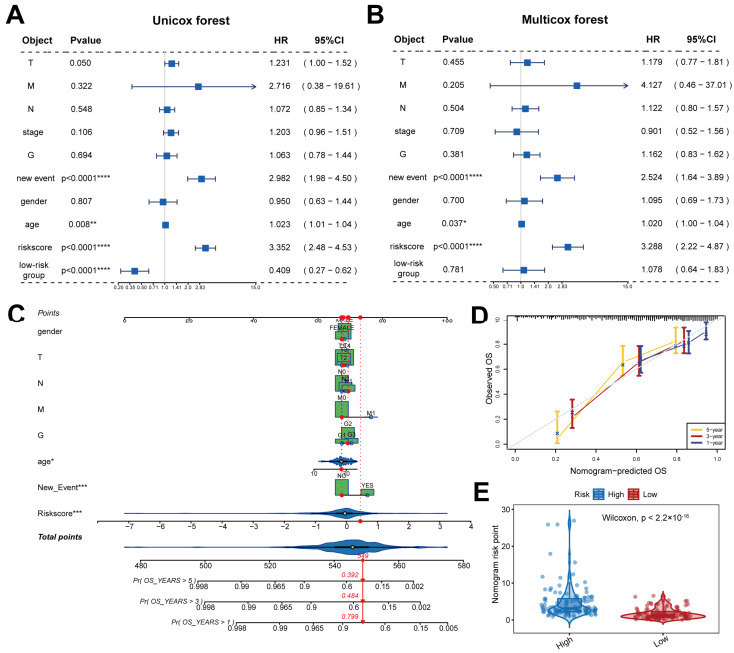
Clinical relevance of the prediction model. (**A**,**B**) Univariate and multivariate Cox regression analyses of clinicopathological variables and the risk score in the OSCC cohort. T, primary tumor classification; M, metastasis classification; N, node classification; G, grading of cancer; HR, hazard ratio. (**C**) Nomogram for predicting overall survival in OSCC patients. (**D**) Calibration curves for 1-, 3-, and 5-year predicted and observed survival. (**E**) Comparison of nomogram risk points between the high- and low-risk groups. * *p* < 0.05, ** *p* < 0.01, *** *p* < 0.001, and **** *p* < 0.0001.

The risk score showed clear associations with the clinicopathological characteristics of OSCC. Survivors had significantly lower risk scores than non-survivors (*p* < 0.0001, Figure 3E). Patients in T3–T4 exhibited higher risk scores than those in T1–T2 (*p* < 0.01, Figure 3F). Similarly, patients in advanced stage (III–IV) had significantly elevated risk scores relative to those in the early stage (I–II) (*p* < 0.05, Figure 3G). In addition, univariate and multivariate Cox regression analyses incorporating clinicopathological parameters and the risk score demonstrated that the risk score served as an independent prognostic factor for OSCC (*p* < 0.0001, Figure 4A,B). The nomogram incorporating the risk score and clinicopathological parameters effectively predicted 1-, 3-, and 5-year survival probabilities, with calibration curves demonstrating good agreement between predicted and observed values (Figure 4C,D). Furthermore, nomogram risk points differed significantly between the high- and low-risk groups (Figure 4E).

### 3.4. The Predictive Value of the LMRG Model for Immunotherapy Response

**Figure 5:**
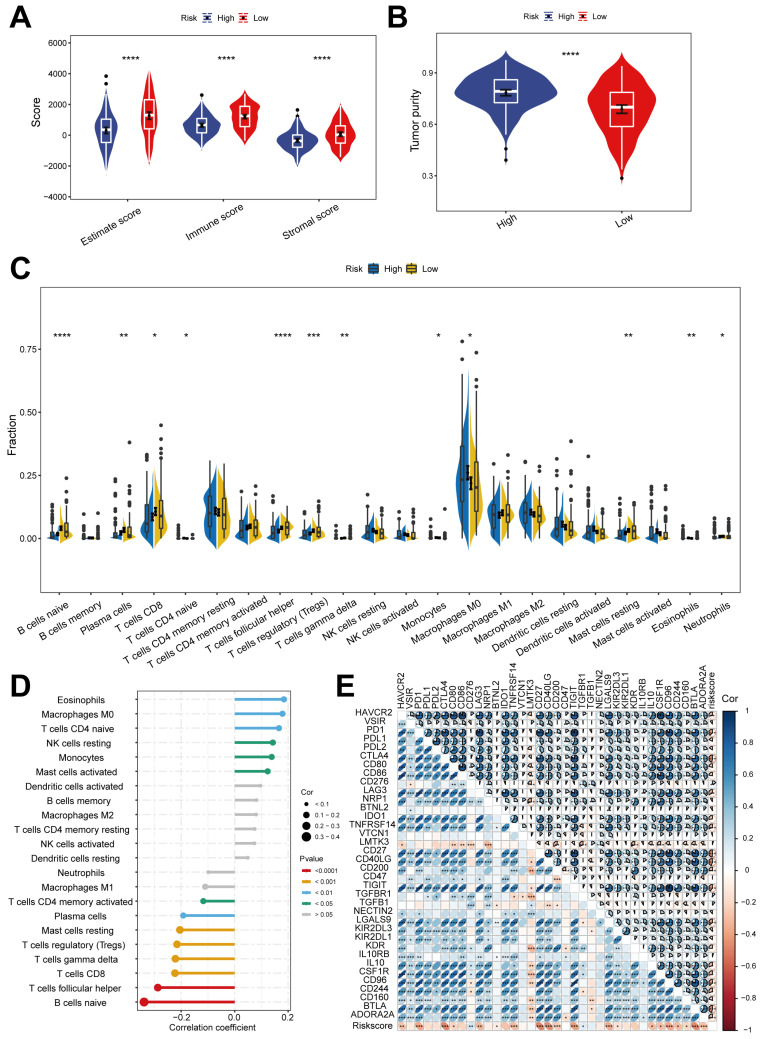
Relationship between the risk score and immune microenvironment in OSCC. (**A**) Comparison of estimate scores, immune scores, and stromal scores between the high-risk and low-risk groups. (**B**) Differences in tumor purity between the two risk groups. (**C**) Infiltration patterns of 22 types of immune cells between the two risk groups. (**D**) Lollipop plots illustrating the correlation between immune cell infiltration and risk scores. (**E**) Heatmap illustrating the correlation between immune checkpoint-related molecules and risk scores. * *p* < 0.05, ** *p* < 0.01, *** *p* < 0.001, and **** *p* < 0.0001.

To investigate the role of risk score in the OSCC immune microenvironment, ESTIMATE analysis was performed. Our data indicated that high-risk patients exhibited lower estimate scores, immune scores, and stromal scores but higher tumor purity (*p* < 0.0001, Figure 5A,B). Concurrently, this high-risk cohort demonstrated a profound reduction in the infiltration of follicular helper T cells and CD8^+^ T cells, whereas M0 macrophage infiltration was markedly elevated (Figure 5C). Lollipop plot analysis confirmed that the risk score was inversely correlated with anti-tumor immune cell abundance (Figure 5D). Furthermore, a widespread negative correlation was observed between the risk score and the expression of most immune checkpoint transcripts (e.g., PD1 and CTLA4). Accordingly, these inhibitory targets showed higher expression in the low-risk subgroup (Figure 5E), suggesting enhanced susceptibility to immune checkpoint blockade in this subgroup.

**Figure 6:**
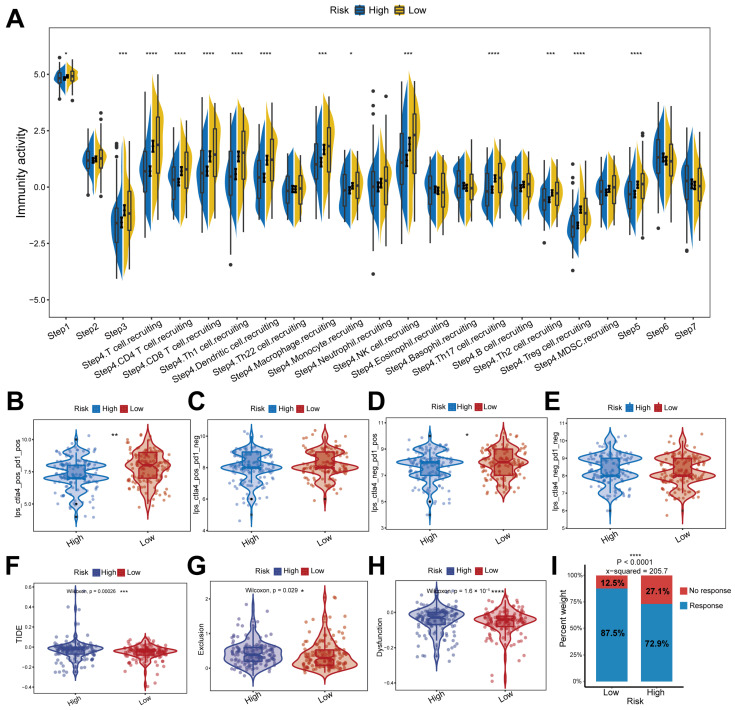
Response to immunotherapy stratified by risk score in OSCC. (**A**) Comparison of immune activity scores between the high-risk and low-risk groups according to TIP analysis. (**B**–**E**) Comparison of IPS scores across four immunotherapy scenarios between the two risk groups. (**F**) Comparison of TIDE scores between the two risk groups. (**G**,**H**) Comparison of immune exclusion and dysfunction scores between the two risk groups. (**I**) Comparison of responsiveness to immune checkpoint therapy between the two risk groups. * *p* < 0.05, ** *p* < 0.01, *** *p* < 0.001, and **** *p* < 0.0001.

To further quantify the functional discrepancy in anti-tumor immunity, we used TIP analysis to calculate immunological activity scores (i.e., anti-tumor immune cycle scores). The low-risk population exhibited significantly higher scores across most steps of the anti-tumor immunological cycle compared to the high-risk group (Figure 6A). Consistently, IPS tracking indicated that low-risk patients responded more favorably to anti-CTLA4 and/or anti-PD1 therapy than high-risk patients (Figure 6B–E). Conversely, TIDE scores were markedly lower in the low-risk subgroup (Figure 6F), suggesting enhanced immunotherapy potential. This was corroborated by reduced dysfunction and exclusion scores within the low-risk subgroup (Figure 6G,H). Ultimately, the predicted immunotherapy response rate was substantially greater in the low-risk subgroup than in the high-risk subgroup (87.5% vs. 72.9%, *p* < 0.0001, Figure 6I).

### 3.5. Mutation Status of Different Risk Groups

**Figure 7:**
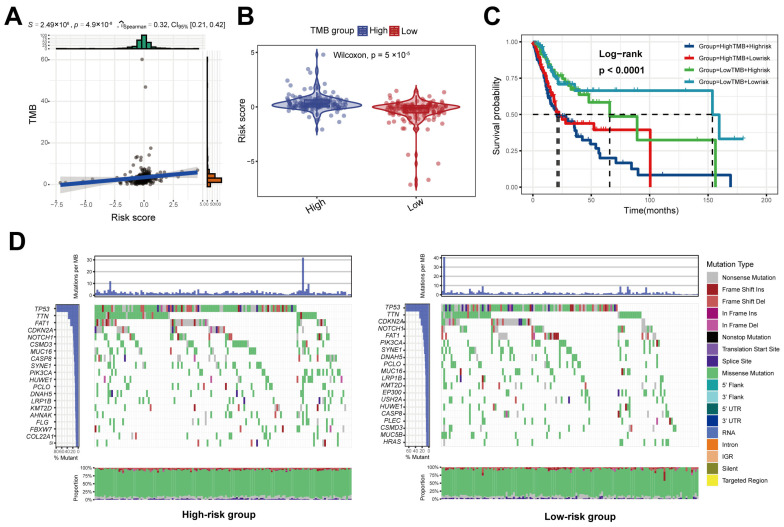
Association between the LMRG risk score and mutation status in OSCC. (**A**) Correlation between TMB and the risk score. (**B**) Differences in the risk score between high-TMB and low-TMB groups. (**C**) Kaplan–Meier survival curves for overall survival across four groups: high TMB + high risk, high TMB + low risk, low TMB + high risk, and low TMB + low risk. (**D**) Waterfall plot of somatic mutations in the high-risk and low-risk groups. Every column corresponds to a single patient. TMB is displayed in the top bar plot. The left bar plot displays the mutation frequency for each gene, with genes ranked by decreasing mutation percentage.

We further investigated the intrinsic association between TMB and the risk score. The results revealed that TMB was positively correlated with the risk score in OSCC (r = 0.32, *p* < 0.0001, Figure 7A), and that patients with high TMB exhibited significantly higher risk scores than those with low TMB (Figure 7B). Furthermore, a combined effect of TMB and the risk score on survival outcomes was observed in patients with OSCC; patients with both high TMB and high risk exhibited worse survival (Figure 7C). Waterfall plots demonstrated differential mutation patterns of the top 20 genes between the two risk groups in OSCC (Figure 7D). Consistent with the TMB findings, patients in the high-risk group exhibited a higher mutation frequency. Specifically, the mutation frequencies of TP53 (high-risk: 78.6% vs. low-risk: 68.5%), TTN (37.1% vs. 28.6%), and FAT1 (29.3% vs. 17.1%) were significantly higher in the high-risk group, whereas CDKN2A mutation frequency (20.0% vs. 24.3%) was elevated in the low-risk group.

### 3.6. Drug Sensitivity Analysis Between Risk Groups

**Figure 8:**
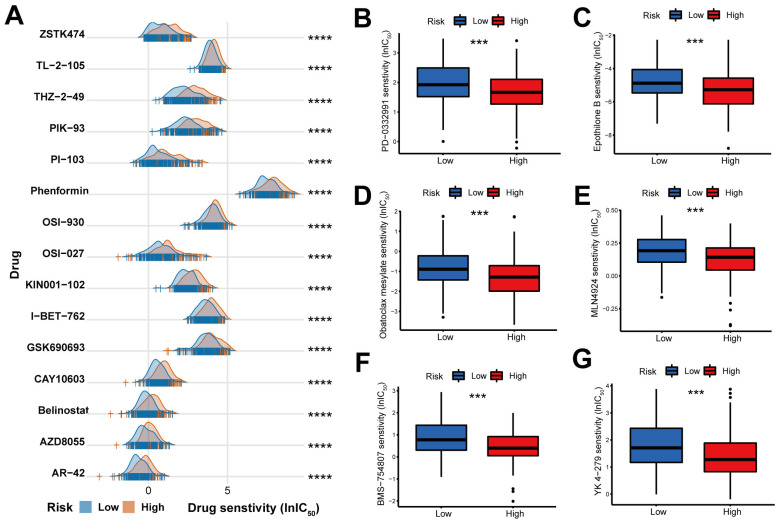
Prediction of drug sensitivity between risk groups. (**A**) Mountain plot delineating drug response profiles between the two risk groups. The top 15 drugs ranked by *p* value are displayed. (**B**–**G**) Predicted drug sensitivity to PD-0332991, Epothilone B, Obatoclax Mesylate, MLN4924, BMS-754807, and YK 4-279 between the two risk groups. *** *p* < 0.001 and **** *p* < 0.0001.

To explore whether the LMRG risk model could guide precision treatment for OSCC, we assessed the predicted ln(IC_50_) values of multiple chemotherapy agents in different risk subgroups. We identified 48 drugs whose ln(IC_50_) values were significantly correlated with the risk scores (Table S3). For the majority of these drugs, including ZSTK474, TL-2-105, and THZ-2-49, the ln(IC_50_) values were significantly higher in the high-risk patients, suggesting that the high-risk group exhibits resistance to these drugs (Figure 8A). Notably, the ln(IC_50_) values for several drugs were significantly lower in the high-risk subgroup, including PD-0332991, epothilone B, obatoclax mesylate, MLN4924, BMS-754807, and YK 4-279 (Figure 8B–G), highlighting potential therapeutic candidates for patients with higher risk scores.

## 4. Discussion

Lipid metabolism dysregulation is widely recognized to be associated with the malignant progression and clinical manifestations of OSCC, especially under the influence of the unique tumor microenvironment and host dietary factors [23,24]. However, clinical prognostic models for OSCC based on lipid metabolism remain scarce. In this study, we developed a novel LMRG risk model for predicting clinical prognosis, immune microenvironment infiltration, immunotherapy efficacy, mutational characteristics, and drug sensitivity in OSCC.

Clinically, OSCC presents significant heterogeneity, making patient prognosis difficult to predict [25,26]. Conventional clinical classification (T, M, N, etc.) relies on microscopic pathological assessment, which overcomes the limitations of macroscopic observation and assists clinicians in evaluating tumor differentiation and grade [27]. However, precise prognosis prediction and personalized treatment of OSCC remain challenging. Traditional clinicopathological parameters have inherent limitations, as histological information alone fails to fully capture the heterogeneity of OSCC [26,28]. In recent years, advances in sequencing technology have enabled genetic profiling to better reflect tumor heterogeneity and stratify patients with distinct prognoses [29,30,31]. In this study, consensus clustering analysis of lipid metabolism genes revealed that the C1 subgroup exhibited a superior prognosis, demonstrating that lipid metabolism patterns can effectively distinguish patient subpopulations. To further facilitate clinical application, we developed an LMRG-based risk model, which calculates individual risk scores based on 16 core lipid metabolism-related genes. Patients with higher risk scores showed markedly poorer survival outcomes, a finding further validated in an independent GEO cohort. We also developed a nomogram integrating the risk score, which may assist clinicians in survival probability estimation and prognostic stratification.

Immunotherapy has recently emerged as a promising therapeutic strategy for patients with OSCC. However, not all patients derive clinical benefit, largely attributable to the complex tumor microenvironment of OSCC [32,33,34]. Lipid metabolism has been implicated in tumor immunity [35,36]. For example, polyunsaturated fatty acids enhance the infiltration of MDSCs via activation of the JAK/STAT3 pathway, and lipid metabolic reprogramming enhances the functional specificity of Treg cells in tumors [36,37]. Given the substantial heterogeneity in immunotherapy responses among patients with OSCC, empirical administration of immunotherapy without prior biomarker assessment may prove ineffective and impose a considerable economic burden [34,38,39]. Therefore, identifying immunotherapy-sensitive patients and delivering targeted interventions represent an urgent clinical need [40]. In this study, the LMRG risk score was found to be closely associated with the abundance of immune cells, such as Treg cells and macrophages, as well as the expression of multiple immune molecules, revealing the pivotal role of lipid metabolism in shaping the tumor immune landscape. More importantly, the LMRG model demonstrated robust predictive value for immunotherapy response. Specifically, high LMRG scores were consistently associated with an immunosuppressive microenvironment, as evidenced by elevated TIDE scores, enhanced T cell dysfunction and exclusion, and decreased IPS, suggesting that patients with high LMRG scores may exhibit resistance to immunotherapy. At the genomic level, the high-risk group exhibited higher TMB and mutation frequencies, particularly in genes such as TP53, TTN, and FAT1. Although elevated TMB has been linked to improved immunotherapy responses in certain cancers [41], this trend was not clearly observed in our cohort. A plausible explanation is that the immunosuppressive tumor microenvironment in OSCC may counteract the immunogenic benefits typically conferred by high TMB [42,43]. Additionally, high TMB was characterized by oncogene activation and tumor suppressor loss, which may further drive tumor progression and reinforce immunosuppression [43].

Drug sensitivity analysis revealed that the high-risk group was less responsive to most chemotherapeutic and targeted agents, suggesting limited efficacy of conventional treatment strategies in this population. Despite this overall resistance pattern, several drugs, including PD-0332991, Epothilone B, Obatoclax mesylate, MLN4924, BMS-754807, and YK 4-279, showed lower predicted IC_50_ values in the high-risk group, suggesting potential therapeutic opportunities. Interestingly, some of these agents may be functionally linked to lipid metabolic processes. For example, CDK4/6 inhibition by PD-0332991 has been reported to influence metabolic regulators involved in lipid synthesis [44], while Obatoclax mesylate and Epothilone B may enhance oxidative stress and lipid peroxidation, processes closely associated with ferroptosis [45,46]. In addition, MLN4924 and YK 4-279 can modulate signaling pathways (e.g., PI3K/AKT pathway) that participate in lipid metabolic regulation [15,47,48,49]. Although not all of these drugs directly target lipid metabolism, these findings suggest that tumors with active lipid metabolic reprogramming may exhibit selective sensitivity to specific agents. Collectively, the LMRG risk model may not only reflect metabolic heterogeneity but also help identify potential vulnerabilities, providing a basis for more tailored treatment strategies in high-risk OSCC patients.

Despite these promising findings, this study has several limitations. First, although the model was validated using independent public datasets, further validation in larger clinical cohorts is required to confirm its robustness and generalizability. Second, the biological mechanisms underlying this lipid metabolism-related signature remain to be fully clarified. In addition, the gene expression data were derived from different platforms (RNA-seq in TCGA and microarray in GEO). Although external validation was performed, such technical heterogeneity prevents direct harmonization of risk score thresholds between cohorts. Future work should incorporate more comprehensive clinicopathological parameters and include experimental investigations to better define the functional roles of genes within the LMRG risk model.

## 5. Conclusions

In conclusion, lipid metabolism genes can effectively stratify OSCC patients into molecular subgroups with distinct prognoses. Moreover, we constructed an LMRG-based risk score model to predict prognosis for OSCC. The risk score was associated with TIME, mutational profile, and drug sensitivity, offering a framework for personalized immunotherapy and chemotherapy strategies.

## Data Availability

The original contributions presented in this study are included in the article/supplementary material. Further inquiries can be directed to the corresponding author.

## Supplementary Materials

The following supporting information can be downloaded at https://www.mdpi.com/article/10.3390/metabo16070455/s1. Table S1. Clinical characteristics of OSCC patients in the TCGA cohort. Table S2. Detailed information of lipid metabolism genes associated with OSCC survival. Table S3. Correlation between drug sensitivity and LMRG risk score.

## Abbreviations

The following abbreviations are used in this manuscript:

AUC	Area Under the Curve
CDF	Cumulative Distribution Function
DEGs	Differentially Expressed Genes
GEO	Gene Expression Omnibus
IPS	Immunophenoscore
LASSO	Least Absolute Shrinkage and Selection Operator
LMRG	Lipid Metabolism-Related Genes
lnIC50	Log-Transformed Half-Maximal Inhibitory Concentration
MDSCs	Myeloid-derived Suppressor Cells
MSigDB	Molecular Signatures Database
OS	Overall Survival
OSCC	Oral Squamous Cell Carcinoma
TCGA	The Cancer Genome Atlas
TIDE	Tumor Immune Dysfunction and Exclusion
TIME	Tumor Immune Microenvironment
TIP	Tracking Tumor Immunophenotype
TMB	Tumor Mutation Burden

## References

[1] Panarese I., Aquino G., Ronchi A., Longo F., Montella M., Cozzolino I., Roccuzzo G., Colella G., Caraglia M., Franco R. (2019). Oral and Oropharyngeal squamous cell carcinoma: Prognostic and predictive parameters in the etiopathogenetic route. Expert Rev. Anticancer. Ther..

[2] Chen Z., Feng X., Pathak J.L., Fu Z., Chen X., Zeng L., Zheng Y., Qiu Q., Qiao L., Wu L. (2026). FAP(+) fibroblasts promote C1QC(+) macrophage infiltration via WNT2 signaling to exacerbate T cell exhaustion in oral squamous cell carcinoma. Cancer Lett..

[3] Tandon P., Dadhich A., Saluja H., Bawane S., Sachdeva S. (2017). The prevalence of squamous cell carcinoma in different sites of oral cavity at our Rural Health Care Centre in Loni, Maharashtra—A retrospective 10-year study. Contemp. Oncol..

[4] Feng X., Zhang C., Chen Z., Chen Z., Zhang Z., Yang S., Liu F., Lee L.H., Wu L., Pathak J.L. (2026). Inhibition of extracellular vesicle secretion by cannabidiol: A promising approach for oral squamous cell carcinoma therapy. Biochem. Pharmacol..

[5] Wang H., Hunter R., Zhang Q., Yu H., Wang J., Yue Y., Geng L., Wu N. (2024). The application of marine polysaccharides to antitumor nanocarriers. Carbohydr. Polym..

[6] Cabral L.G.S., Martins I.M., Paulo E.P.A., Pomini K.T., Poyet J.L., Maria D.A. (2025). Molecular Mechanisms in the Carcinogenesis of Oral Squamous Cell Carcinoma: A Literature Review. Biomolecules.

[7] Wu C.S., Li H.P., Hsieh C.H., Lin Y.T., Yi-Feng Chang I., Chung A.K., Huang Y., Ueng S.H., Hsiao Y.C., Chien K.Y. (2025). Integrated multi-omics analyses of oral squamous cell carcinoma reveal precision patient stratification and personalized treatment strategies. Cancer Lett..

[8] Maharajan N., Benyamien-Roufaeil D.S., Brown R.A., Portney B.A., Banerjee A., Zalzman M. (2025). Cancer stem cell mechanisms and targeted therapeutic strategies in head and neck squamous cell carcinoma. Cancer Lett..

[9] Cao L.Q., Xie Y., Fleishman J.S., Liu X., Chen Z.S. (2024). Hepatocellular carcinoma and lipid metabolism: Novel targets and therapeutic strategies. Cancer Lett..

[10] Saxton R.A., Sabatini D.M. (2017). mTOR Signaling in Growth, Metabolism, and Disease. Cell.

[11] Halma M.T.J., Tuszynski J.A., Marik P.E. (2023). Cancer Metabolism as a Therapeutic Target and Review of Interventions. Nutrients.

[12] Röhrig F., Schulze A. (2016). The multifaceted roles of fatty acid synthesis in cancer. Nat. Rev. Cancer.

[13] Wang Y., Zhang X., Wang S., Li Z., Hu X., Yang X., Song Y., Jing Y., Hu Q., Ni Y. (2022). Identification of Metabolism-Associated Biomarkers for Early and Precise Diagnosis of Oral Squamous Cell Carcinoma. Biomolecules.

[14] Maan M., Peters J.M., Dutta M., Patterson A.D. (2018). Lipid metabolism and lipophagy in cancer. Biochem. Biophys. Res. Commun..

[15] Xiao Q., Xia M., Tang W., Zhao H., Chen Y., Zhong J. (2024). The lipid metabolism remodeling: A hurdle in breast cancer therapy. Cancer Lett..

[16] Li D., Li Y. (2020). The interaction between ferroptosis and lipid metabolism in cancer. Signal Transduct. Target. Ther..

[17] Liu Y., Zhao Y., Song H., Li Y., Liu Z., Ye Z., Zhao J., Wu Y., Tang J., Yao M. (2024). Metabolic reprogramming in tumor immune microenvironment: Impact on immune cell function and therapeutic implications. Cancer Lett..

[18] Lim S.A., Wei J., Nguyen T.M., Shi H., Su W., Palacios G., Dhungana Y., Chapman N.M., Long L., Saravia J. (2021). Lipid signalling enforces functional specialization of T(reg) cells in tumours. Nature.

[19] Ma H., Liu C., Li X., Zuo L., Li C., Xu X., Zhang S., Ma X., Yue E., Qiao B. (2025). Lipid metabolites as biomarkers and therapeutic targets in oral squamous cell carcinoma. BMC Oral. Health.

[20] Sakurai K., Tomihara K., Yamazaki M., Tanuma J.I., Yamada S.I. (2025). High CD36 Expression Predicts Aggressive Invasion and Recurrence in Oral Squamous Cell Carcinoma. Int. J. Mol. Sci..

[21] Chen X., Liu Q., Chen Y., Wang L., Yang R., Zhang W., Pan X., Zhang S., Chen C., Wu T. (2022). Carboxylesterase 2 induces mitochondrial dysfunction via disrupting lipid homeostasis in oral squamous cell carcinoma. Mol. Metab..

[22] Bradley A., Van Der Meer R., McKay C.J. (2019). A systematic review of methodological quality of model development studies predicting prognostic outcome for resectable pancreatic cancer. BMJ Open.

[23] Hu Q., Peng J., Chen X., Li H., Song M., Cheng B., Wu T. (2019). Obesity and genes related to lipid metabolism predict poor survival in oral squamous cell carcinoma. Oral Oncol..

[24] Zhang J., Peng J., Wang S., Wang L., Sun Y., Xia J., Cheng B., Hu Q. (2025). Perilipin2-dependent lipid droplets accumulation promotes metastasis of oral squamous cell carcinoma via epithelial-mesenchymal transition. Cell Death Discov..

[25] Zhang Q., Ding L., Li J., Liu K., Xia C., Chen S., Huang X., Pu Y., Song Y., Hu Q. (2024). Single-cell RNA sequencing of OSCC primary tumors and lymph nodes reveals distinct origin and phenotype of fibroblasts. Cancer Lett..

[26] Shang Q., Jiang Y., Wan Z., Peng J., Xu Z., Li W., Yang D., Zhao H., Xu X., Zhou Y. (2024). The clinical implication and translational research of OSCC differentiation. Br. J. Cancer.

[27] Almangush A., Mäkitie A.A., Triantafyllou A., de Bree R., Strojan P., Rinaldo A., Hernandez-Prera J.C., Suárez C., Kowalski L.P., Ferlito A. (2020). Staging and grading of oral squamous cell carcinoma: An update. Oral Oncol..

[28] Liu Z., Zhang Z., Zhang Y., Zhou W., Zhang X., Peng C., Ji T., Zou X., Zhang Z., Ren Z. (2024). Spatial transcriptomics reveals that metabolic characteristics define the tumor immunosuppression microenvironment via iCAF transformation in oral squamous cell carcinoma. Int. J. Oral Sci..

[29] Ravindran S., Ranganathan S., Karthikeyan R., Nandini J., Shanmugarathinam A., Kannan S.K., Prasad K.D., Marri J., Rajaganapathi K. (2025). The role of molecular biomarkers in the diagnosis, prognosis, and treatment stratification of oral squamous cell carcinoma: A comprehensive review. J. Liq. Biopsy.

[30] Sun Y., Cheng G., Wei D., Luo J., Liu J. (2024). Integrating omics data and machine learning techniques for precision detection of oral squamous cell carcinoma: Evaluating single biomarkers. Front. Immunol..

[31] Chen C., Zhang Y., Liu Y., Hang L., Yang J. (2022). Expression of Tumor Suppressor SFRP1 Predicts Biological Behaviors and Prognosis: A Potential Target for Oral Squamous Cell Carcinoma. Biomolecules.

[32] Liu H.M., Xiong X.P., Yu Z.L., Shao Z., Chen G.L., Liu Y.T., Wang X.X., Fu Q.Y., Cheng X.X., Li J. (2025). Neoadjuvant immunotherapy with or without chemotherapy in locally advanced oral squamous cell carcinoma: Randomized, two-arm, phase 2 trial. Cell Rep. Med..

[33] Lei X., Lei Y., Li J.K., Du W.X., Li R.G., Yang J., Li J., Li F., Tan H.B. (2020). Immune cells within the tumor microenvironment: Biological functions and roles in cancer immunotherapy. Cancer Lett..

[34] Ju W.T., Xia R.H., Zhu D.W., Dou S.J., Zhu G.P., Dong M.J., Wang L.Z., Sun Q., Zhao T.C., Zhou Z.H. (2022). A pilot study of neoadjuvant combination of anti-PD-1 camrelizumab and VEGFR2 inhibitor apatinib for locally advanced resectable oral squamous cell carcinoma. Nat. Commun..

[35] Cui M.Y., Yi X., Cao Z.Z., Zhu D.X., Wu J. (2022). Targeting Strategies for Aberrant Lipid Metabolism Reprogramming and the Immune Microenvironment in Esophageal Cancer: A Review. J. Oncol..

[36] Wang G., Xu J., Zhao J., Yin W., Liu D., Chen W., Hou S.X. (2020). Arf1-mediated lipid metabolism sustains cancer cells and its ablation induces anti-tumor immune responses in mice. Nat. Commun..

[37] Yan C., Zheng L., Jiang S., Yang H., Guo J., Jiang L.Y., Li T., Zhang H., Bai Y., Lou Y. (2023). Exhaustion-associated cholesterol deficiency dampens the cytotoxic arm of antitumor immunity. Cancer Cell.

[38] Tang T., Huang X., Zhang G., Hong Z., Bai X., Liang T. (2021). Advantages of targeting the tumor immune microenvironment over blocking immune checkpoint in cancer immunotherapy. Signal Transduct. Target. Ther..

[39] Couchoud C., Fagnoni P., Aubin F., Westeel V., Maurina T., Thiery-Vuillemin A., Gerard C., Kroemer M., Borg C., Limat S. (2020). Economic evaluations of cancer immunotherapy: A systematic review and quality evaluation. Cancer Immunol. Immunother..

[40] Ma Q., Ren J., Wang R., Yuan Y., Tao X. (2024). Predicting response to immunotherapy in oral squamous cell carcinoma via a CT-based radiomics model. BMC Med. Imaging.

[41] Song W., Ren J., Xiang R., Kong C., Fu T. (2021). Identification of pyroptosis-related subtypes, the development of a prognosis model, and characterization of tumor microenvironment infiltration in colorectal cancer. Oncoimmunology.

[42] Lei M., Luo C., Zhang J., Cao W., Ge J., Zhao M. (2022). A m(6)A methyltransferase-mediated immune signature determines prognosis, immune landscape and immunotherapy efficacy in patients with lung adenocarcinoma. Cell. Oncol..

[43] Gu W., Kim M., Wang L., Yang Z., Nakajima T., Tsushima Y. (2021). Multi-omics Analysis of Ferroptosis Regulation Patterns and Characterization of Tumor Microenvironment in Patients with Oral Squamous Cell Carcinoma. Int. J. Biol. Sci..

[44] Rodencal J., Kim N., He A., Li V.L., Lange M., He J., Tarangelo A., Schafer Z.T., Olzmann J.A., Long J.Z. (2024). Sensitization of cancer cells to ferroptosis coincident with cell cycle arrest. Cell Chem. Biol..

[45] Nguyen M., Cencic R., Ertel F., Bernier C., Pelletier J., Roulston A., Silvius J.R., Shore G.C. (2015). Obatoclax is a direct and potent antagonist of membrane-restricted Mcl-1 and is synthetic lethal with treatment that induces Bim. BMC Cancer.

[46] Li F., Huang T., Tang Y., Li Q., Wang J., Cheng X., Zhang W., Zhang B., Zhou C., Tu S. (2021). Utidelone inhibits growth of colorectal cancer cells through ROS/JNK signaling pathway. Cell Death Dis..

[47] Ge M., Huang L., Ma Y., Sun S., Wu L., Xu W., Yang D. (2022). MLN4924 Treatment Diminishes Excessive Lipid Storage in High-Fat Diet-Induced Non-Alcoholic Fatty Liver Disease (NAFLD) by Stimulating Hepatic Mitochondrial Fatty Acid Oxidation and Lipid Metabolites. Pharmaceutics.

[48] Yu L., Wu X., Chen M., Huang H., He Y., Wang H., Li D., Du Z., Zhang K., Goodin S. (2017). The Effects and Mechanism of YK-4-279 in Combination with Docetaxel on Prostate Cancer. Int. J. Med. Sci..

[49] Xiong S., Huang W., Liu X., Chen Q., Ding Y., Huang H., Zhang R., Guo J. (2022). Celecoxib Synergistically Enhances MLN4924-Induced Cytotoxicity and EMT Inhibition via AKT and ERK Pathways in Human Urothelial Carcinoma. Cell Transplant..

